# Perceptions of shared care among survivors of colorectal cancer from non-English-speaking and English-speaking backgrounds: a qualitative study

**DOI:** 10.1186/s12875-018-0822-6

**Published:** 2018-07-30

**Authors:** Lawrence Tan, Gisselle Gallego, Thi Thao Cam Nguyen, Les Bokey, Jennifer Reath

**Affiliations:** 1 Department of General Practice, School of Medicine, Western Sydney University, Locked Bag 1797, Penrith, NSW 2751 Australia; 2 School of Medicine, University of Notre Dame, 140 Broadway, Sydney, NSW 2007 Australia; 3 Bonnyrigg Family Medical Centre, Bonnyrigg, NSW Australia; 4 Department of Surgery, Western Sydney University, Locked Bag 1797, Penrith, NSW 2751 Australia

**Keywords:** Colorectal cancer, Survivorship, Care coordination, Cultural and linguistic diversity, General practice

## Abstract

**Background:**

Colorectal cancer (CRC) survivors experience difficulty navigating complex care pathways. Sharing care between GPs and specialist services has been proposed to improve health outcomes in cancer survivors following hospital discharge. Culturally and Linguistically Diverse (CALD) groups are known to have poorer outcomes following cancer treatment but little is known about their perceptions of shared care following surgery for CRC. This study aimed to explore how non-English-speaking and English-speaking patients perceive care to be coordinated amongst various health practitioners.

**Methods:**

This was a qualitative study using data from face to face semi-structured interviews and one focus group in a culturally diverse area of Sydney with non-English-speaking and English-speaking CRC survivors. Participants were recruited in community settings and were interviewed in English, Spanish or Vietnamese. Interviews were recorded, transcribed, and analysed by researchers fluent in those languages. Data were coded and analysed thematically.

**Results:**

Twenty-two CRC survivors participated in the study. Participants from non-English-speaking and English-speaking groups described similar barriers to care, but non-English-speaking participants described additional communication difficulties and perceived discrimination. Non-English-speaking participants relied on family members and bilingual GPs for assistance with communication and care coordination. Factors that influenced the care pathways used by participants and how care was shared between the specialist and GP included patient and practitioner preference, accessibility, complexity of care needs, and requirements for assistance with understanding information and navigating the health system, that were particularly difficult for non-English-speaking CRC survivors.

**Conclusions:**

Both non-English-speaking and English-speaking CRC survivors described a blend of specialist-led or GP-led care depending on the complexity of care required, informational needs, and how engaged and accessible they perceived the specialist or GP to be. Findings from this study highlight the role of the bilingual GP in assisting CALD participants to understand information and to navigate their care pathways following CRC surgery.

**Electronic supplementary material:**

The online version of this article (10.1186/s12875-018-0822-6) contains supplementary material, which is available to authorized users.

## Background

Colorectal cancer (CRC) is the second-largest cause of cancer death in Australia. The five-year survival rate has risen from 48% in 1983–1987, to 64% in 2008–2012 [1]. Proposals have been made for the care of cancer survivors to be shared with general practitioners (GPs) rather than being exclusively specialist-centred. Hospital-based care tends to focus on detecting disease recurrence, while care shared with primary care services could potentially improve psychosocial support, care for other comorbidities, and preventive care [2, 3].

Cancer survivors have described multiple care needs following surgery, [4–6] and poor coordination of health care is a recurrent theme in cancer survivorship research [7]. A randomised controlled trial conducted in Australia comparing CRC follow up in general practice with surgical based follow-up showed no difference in patient satisfaction, detection of recurrence or mortality [8]. However cancer survivors, GPs and specialists have mixed feelings about shared care [9]. Some studies show patients are more satisfied with specialist care [10] even though shared care was found to be more cost-effective [11]. Elsewhere, patients were satisfied overall with follow-up in primary care unless they had more challenges in recovery, when the organisation of care became “complex and variable” [12]. They valued support from an “active” GP, and also reassurance from their specialists [13]. Concerns mentioned by GPs regarding participating in shared care for colorectal cancer survivors included time, cost, poor communication and inadequate transfer of information between specialist and GP settings [14–16].

Australia is a multicultural country with almost half of its population (49%) born overseas. According to the 2016 Census more than one-fifth (21%) of Australians spoke a language other than English at home [17]. This is important considering that people from minority culturally and linguistically diverse (CALD) backgrounds have lower screening rates, poorer cancer outcomes [18, 19] and greater informational needs [20]. For example, Caucasian cancer survivors in the United States of America (USA) were more likely to have follow-up screening, preventive care, access to mental health services and more frequent visits to their physician compared with patients from other ethnicities [21].

In Australia, patients can freely choose their GP. If required, the GP refers them to a private specialist, often taking into account the patient’s preferences when selecting which specialist to refer to. Patients who go through the public hospital system have no choice of specialist. Public hospitals are free and visits to GPs (who provide community based primary health care) and community based specialists are cost-subsidised through a universal health insurance (Medicare). Private insurance is also available for those who choose and can afford to purchase it, providing additional subsidies for private hospital access and choice of specialist. Cancer follow up is provided across all these settings, but no formal model of shared care for CRC survivors is currently in place. Little research has been undertaken on cancer survivorship in CALD communities in the Australian setting [22]. This study aimed to explore how CRC survivors from CALD backgrounds who speak languages other than English at home, as well as those from English-speaking backgrounds, perceive care to be coordinated amongst various health practitioners in an Australian setting.

## Methods

Qualitative methodology was chosen to explore the experiences and perceptions of the participants [23] in order to help the researchers understand how they navigated the health system to overcome barriers to care, which relates to how they felt their care was being coordinated.

### Setting

This qualitative study was undertaken between 2015 and 2016, mainly in South West Sydney. South West Sydney is known for its cultural diversity with 45% of the population speaking a language other than English at home. After Arabic, Vietnamese is the second-most common non-English language spoken at home. The next most frequently spoken languages are Mandarin, Cantonese and Hindi; Spanish being the tenth-most frequently spoken language [24].

### Participants and recruitment

In order to represent a continuum of experiences, stratified purposive sampling [25] was used to recruit participants 6 weeks to 8 years following CRC surgery, who spoke either English, Spanish or Vietnamese at home. These three languages were spoken by members of the research team. We used the language spoken at home as a proxy for the cultural background participants identified with. Participants were recruited from community support groups, and GP and colorectal surgeon private rooms. None of the GPs treating the patients at the time of the CRC diagnosis and initial follow-up were involved in this research. Radio and newspaper announcements were made in the target languages. Potential participants were asked to contact the researchers. They were then informed verbally about the aim of the study and provided with a printed participant information statement that explained the aims of the research, and a consent form in their preferred language. These forms had been translated by translators from the National Accreditation Authority for Translators and Interpreters. We set no formal exclusion criteria although one potential participant was excluded because he was still recovering in hospital, and another because she had a bowel resection during her breast cancer treatment, but did not actually have CRC. Participants were offered a small gift voucher as a token of appreciation for their time.

### Data collection

Participants were given the option of a face-to-face or telephone interview, or participation in a focus group. All participants provided written informed consent except for two who were interviewed by phone, whose verbal consent to participate in the study was recorded after the consent form was read out to them as approved by the ethics committee. An interview guide with open-ended questions and prompts was prepared in English, Spanish and Vietnamese (Additional file 1). The interview guide was developed following a review of the literature and discussion within the research team. LT, a male GP researcher, is fluent in English and Spanish and conducted the Spanish interviews and some of the English interviews. TN, a female GP researcher, is a native speaker of Vietnamese and conducted some of the Vietnamese interviews. The remaining English and Vietnamese interviews were conducted by research assistants (a male and a female medical student trained in qualitative research interviewing, who are native speakers of both English and Vietnamese). In order to respect cultural preferences, Vietnamese interviews were conducted where possible by researchers of the same gender as the participants. Participants were asked to speak in their preferred language. Interviews were conducted in the patient’s home, GP surgery, coffee shop or by telephone according to participant preference. Five participants were accompanied by a family member – one English-speaker, one Spanish-speaker and three Vietnamese-speakers. Interviews took between 30 to 45 min and concurrent field notes were taken. The focus group with three English-speaking participants was facilitated by LT together with a research assistant in a meeting room at a cancer survivor centre, that lasted for approximately 1 hour. Interviews and the focus group session were digitally recorded and transcribed in the original language. Participants were offered a copy of the transcripts to check. Transcriptions were performed by a professional transcription service in the original language and their integrity checked by the interviewer. Transcripts were analysed concurrently with the interviewing, which continued until no new themes emerged, suggesting data saturation [26] within the groups of participants studied.

### Data analysis

Participants were de-identified and transcripts labelled with an individual number and letters denominating the gender and language(s) spoken. Vietnamese transcripts were translated by the bilingual interviewers. Spanish transcripts were analysed by LT and GG, who cross-checked their translations with each other and translated relevant excerpts into English for review by the research team. GG is a native speaker of Spanish who is fluent in English. A thematic analysis was conducted [26]. LT read and reread the transcripts and applied descriptive codes to the data. Initially, early transcripts were reviewed and coded independently by GG and JR and a tentative coding framework was agreed by all three researchers. This framework was applied to subsequent interview transcripts by LT and GG, and the framework reviewed and revised at regular team meetings including JR until all 22 interviews had been coded using the final agreed framework. At a later stage the codes were grouped into themes derived from the data. Illustrative quotes not in English were translated by the research assistants, TN and LT, then checked by a second native speaker of that language. Data were managed using Microsoft® Excel® 2016. Ethics approval was provided by the Western Sydney University Human Research Ethics Committee (approval number H9067).

## Results

Twenty-two patients participated, of whom 10 spoke English at home, five spoke Spanish and four spoke Vietnamese. Two were bilingual Spanish-English, and one was bilingual Vietnamese-English. The three bilingual participants preferred to be interviewed in English. All the Vietnamese-speakers had been born in Vietnam and had migrated to Australia between 29 to 37 years ago. The Spanish-speakers came from Argentina, Chile, El Salvador and Uruguay. They had lived in Australia for 23 to 40 years. Three native English-speakers participated in a focus group and the remainder were interviewed individually. The English-speakers were all born in Australia or the United Kingdom, except for three who had been born in Croatia, Lebanon and the Netherlands. These three had come to Australia in childhood. In our study, most of the English-speaking participants had private insurance, while most of the Spanish-speaking ones did not have private insurance and used the public hospital service. Half of the Vietnamese-speakers had private health insurance. See Table 1 for other participant characteristics.

**Table 1:** Participant characteristics (*n* = 22)

	Mean	Range
Age, years	65	38–88
Years since surgery	2.5	0.1–8
	N	%
Female	12	54.5
Relative present at interview	5	22.7
Private health insurance	14	63.6
Language spoken at home		
English	10	45
Spanish	5	23
Vietnamese	4	18
English-Spanish	2	9
English-Vietnamese	1	5
Cancer treatment		
Surgery only	7	32
Surgery plus chemotherapy	7	32
Surgery plus neoadjuvant therapy	4	18
Surgery plus radiotherapy	1	5
Surgery including colostomy	11	50

Participants described a complex blend of barriers to care, strategies used to meet their care needs and discussed how they perceived care to be coordinated between their care providers. Table 2 summarises the themes and subthemes identified.

**Table 2:** Colorectal cancer survivorship themes

Barriers to Care	Cost
	Logistics
	Timeliness
	Language and communication
	Perceived discrimination
Strategies used to meet care needs	Family members
	Bilingual GP
	Private health insurance
Coordination of care	Variability in information flow
	Variability in GP and specialist involvement
	Role of the bilingual GP

### Barriers to care

Participants described numerous difficulties receiving the care they required, including getting appointments, navigating care pathways, understanding information, perceived discrimination, cost and logistical difficulties.

Both English-speaking and non-English-speaking participants found specialist and GP appointments could take a long time, but in general it was easier to get an appointment with a GP:

“*It would always take a number of weeks to get to see [the specialist] because he was heavily booked*” (Male English-speaker).

Another barrier common to both groups was confusion about who to go to when care was required after hours:

*“I ring up the hospital and ask them what I should do if I have this problem, they say, first of all you need to go to your GP doctor to let him have a look how serious is it. If that serious he would send - give you the letter referred you to hospital… at the time it's six o'clock, your GP doctor closed, what you going to do?”* (Male bilingual Vietnamese-English speaker)

Communication was a particular barrier for non-English-speaking participants. They were sometimes unclear about what was being treated and what treatment had been given. For example, one of the Vietnamese-speaking participants described how she did not even know what surgery had been performed:

*“[I] would look at the scar and try and guess what happened”.* (Female Vietnamese-speaker)

They were also unsure where or when to attend for follow-up, who to consult if problems arose, or what support services were available in the community. A bilingual English-Vietnamese speaker expressed both his difficulty understanding and his difficulty communicating in English, even though he chose to speak in that language:

*“… but who will be the responsibility to help you, to understand what you do for first month, second month, third month, I don't know. For myself or for whoever Western, they can read it in English or they can understand thing would be good, but for traditional like, for my mum and if you don't have relationship with English I don't know what will be difficult for them”* [sic].

Even though interpreters were available in hospital to assist with communication, some participants were reluctant to impose on interpreters’ time.

*“There were interpreters but I think that …when you ask too many questions it feels like they don’t like it”* (Male Vietnamese-speaker)

Two of the five Vietnamese-speaking patients also described perceived discrimination, compounded by an inability to express themselves in English:

*“The way she put the needle in was as if she thought of me like a dog or an animal! It was very painful but I couldn’t say a word because no one understood my language - Vietnamese. Therefore, I told her that I am in pain in Vietnamese ‘*dau’ *and I stared at her”* (Female Vietnamese-speaker).

One described this as double-discrimination – because of his race, and also because of his inability to speak English:

*“Asian people are already discriminated against and not being able to speak English; then those two factors combine”* (Male Vietnamese-speaker).

He felt this contributed to a delay in diagnosis of a serious post-operative complication because it appeared to him that staff would not pay attention to his complaints of pain.

Many participants in both English-speaking and non-English-speaking groups described difficulty accessing their specialists because of cost. Other logistical issues included difficulty finding parking, and having to make sure they didn’t eat or drink too much that day because of a lack of toilet facilities at the specialist’s consulting rooms:

“*You have one 15-minute session with your surgeon, and once that 15 minutes is up, there’s the door for $100 and the next patient comes in*” (Male English-speaker)

### Strategies used to meet care needs

Non-English-speaking participants brought family members with them to specialist appointments, for emotional support as well as to interpret for them:

*“Someone always goes with me, always”* (Female Spanish-speaker).

Interestingly, an English-speaking participant who was hearing-impaired used the same strategy:

“… *So I'm her second pair of ears*” (sister of female English-speaker)

All non-English-speaking participants in our study had a bilingual GP whom they consulted. The bilingual GP assisted non-English-speaking participants by providing information and advice on how to navigate the health care system:

*“Well if I didn't feel well in myself or something like that, then I would call my son to drive me to the [Vietnamese-speaking] family doctor. I could ask him this and that, and then he would direct me to who I should call or where I should go”* (Female Vietnamese-speaker).

One Spanish-speaking female participant described consulting a GP who only spoke English when her usual bilingual GP was unavailable. On this occasion she brought her daughter to the consultation to interpret for her.

Both non-English-speaking and English-speaking participants perceived private health insurance to facilitate in their cancer management by reducing the waiting time, providing additional private hospital benefits such as permission for family to stay over, and improved access to the specialist.

“*I paid [for the colonoscopy in a private hospital], because the specialist said that if you wait [for a public hospital colonoscopy] it can take quite a while ... and I also bought medical insurance”* (Female Vietnamese-speaker)

### Coordination of care

Participants described a wide range of health professionals involved in their care, including stoma nurses, physiotherapists, psychologists and even a surgeon’s receptionist who would assist by ringing up for appointments with other specialists. None of the participants described a formal shared care arrangement between specialist and primary care services, nor mentioned a written shared care plan. They described a variable information flow between their health providers, and tended to seek help from the health provider the participant felt was most involved or appropriate to meet their needs. Some participants assumed specialists and GPs were in communication, while others reported poor information flow:

*“Yeah, and nothing's linked ... three different hospitals I've got to go to ... you've got to explain yourselves so many times, over and over again”* (Female English-speaker)

Sometimes non-English-speaking and English-speaking participants or their relatives, would take the initiative to assist in the information flow:

“*So I ended up trying to collect everything and I got something good off - the first person who had written up the story was the oncologist, I said 'Oh, can I have a copy of that, because that's got everything?’ … So it took me a while to get it all together and say 'OK, the person who has got to have this is my GP. And I've got to stick to this one GP’*” (Female English-speaker).

Both English-speaking and non-English-speaking background participants had a high degree of trust in their private specialist, particularly when there were complex care needs and they needed someone to coordinate everything:

*“I wasn't really searching for too much information, I just basically left it in the hands of the Professor and his staff*” (Male English speaker)

“*Up front we asked [the private surgeon], we wanted somebody to be the manager of the whole thing and not have to talk to different doctors and wonder who is doing what.*” (Male English-speaker).

On the other hand, one participant felt devastated, starting to cry as she described how her oncologist seemed dismissive of her mental health concerns. Another felt rejected by the surgeon when seeking help for persistent diarrhoea following surgery:

“*But afterwards I felt a lot of rejection; I went to complain, because I felt as if he didn’t want to know anything more about it. He was washing his hands. That’s how I felt.*” (Male Spanish-speaker)

Some English-speaking participants had little or no relationship with their GPs, with one not even recalling the GP’s name. Following the initial diagnosis and referral, some English-speaking participants viewed GPs as irrelevant, only becoming involved in care after they had been discharged by the specialist:

“*I don't know what I would expect from a GP afterwards, well, I haven't got a lot, so I don't, but I don't know what they can do. You've had the treatment, haven't you, and you're moving on, so there's not a lot they can do except keep an eye on you and I guess after I get cut adrift after these five years they'll take more of an active role”* (Male English-speaker).

Other English-speaking patients only consulted GPs for non-cancer matters, although provision of preventive care was not mentioned:

“*I've seen my GP for, I think I've just had to get a script for my blood pressure tablets and so on and so forth*” (Male English-speaker)

For CRC-related issues they preferred to consult the surgeon, and sometimes the GP also appeared to defer to the surgeon:

*“But anything more detailed, he would say 'of course, this is a matter be addressed by your surgeon,' which is easier said than done when you've got to wait three and a half weeks to get an early appointment”* (Male English-speaker)

Other participants described their GP as being engaged and pro-active in managing the cancer recovery process. One detected a post-operative wound infection when asked to make a house-call because of an unrelated issue. In this case the participant had not even been told to see the GP after the surgery:

“*But that was for the ear he came, and that's when he says, 'I'll look at your wound while I'm here' [laughs]. So that's how. But they didn't say to me that I had to see my GP; they just said I had to see [the gastroenterologist] within 2-3 weeks*” (Female English-speaker).

In another situation an involved GP was seen to provide good ongoing whole-person care when the participant felt specialist care was fragmented and did not address all her needs:

“*You didn't realise how much you needed to rely on a GP for all the referrals and just to have somebody who collected all the information that came in, knew your history and could refer you to different people and if you came in and said 'look, I'm really low today and not doing well,' to know where you were at, to know where the medication was at and what to do and yeah, just to be that go-to person*” (Female English-speaker).

In comparison with the English-speaking participants, bilingual GPs were perceived by many non-English-speaking participants to be key coordinators of care who could support the participant in their own language. These participants displayed a high level of trust in their bilingual GPs who could also explain what the follow-up arrangements were, and facilitate communication between the participant and the specialist or hospital without requiring assistance from family members or official interpreters:

“*I would say [to the surgeon], ‘Please, you have to write to my father’s doctor, please you have to send him all the information because I don’t go with him, because the [GP] speaks Spanish’*” (daughter of male Spanish-speaker)

*“Like, I felt that the [Vietnamese-speaking] GP was important because if anything happened, I would have to go get checked by the GP. I could talk to the GP, call the GP, if I needed medication or had any troubles. I would have to tell the GP and the GP could contact the specialists. They all worked together.”* [Female Vietnamese-speaker]

## Discussion

English-speaking and non-English-speaking CRC survivors had a similar range of barriers to care, but non-English-speaking patients faced additional difficulties due to communication issues and perceived discrimination. Participants in this study did not describe formal shared care arrangements, but described using GP-led, specialist-led or shared models of care depending on multiple factors such as ease of access, private insurance, presence of co-morbidities, complexity of care, and perceived interest or engagement by the GP or specialist. Bilingual GPs were described by participants as being key in coordinating care for participants who came from a non-English-speaking background.

The Primary Care Collaborative Cancer Clinical Trials Groups’ principles statement on implementing shared care of cancer patients [27] emphasises that shared care should be acceptable and flexible; with clear expectations, communication pathways, implementation process, integration with existing processes and evaluation. Participants in this study did not always have expectations of shared care clarified, and mostly assumed the specialist and GP communicated with one another. Other studies have provided a different perspective from GPs who identified issues with information flow [28]. In our study, where communication was perceived to be deficient, participants or their carers often took steps to ensure their GP received the information, or took charge of the information themselves by asking for copies so they could show them to the next health professional consulted. Examples of deficiencies in care from our study included one participant developing a wound infection after the surgery, who had not been told to see her GP for post-operative review; another whose medication changes had not been communicated with the GP; and others who described hospital results not being available at the time of post-surgical consultation with the GP.

Specialist-led care rather than GP-led care was demonstrated in both non-English-speaking and English-speaking groups, particularly if they had more complicated cancer treatment, fewer co-morbidities, and easy access to specialist care. Participants who had private insurance in our study had a greater reliance on specialist-led care, similar to a previous study on patient experiences of the referral process for CRC [29]. Some participants considered GPs to be irrelevant in the care of cancer survivors until they had been discharged by the specialist. In other cases, GPs themselves appeared to disengage from cancer survivor care, influencing the participant to seek specialist help.

On the other hand, GP-led care was perceived by participants in our study to be more important when participants had physical and emotional needs not addressed by specialists. Studies of Danish, US and Australian GPs similarly showed they were consulted by CRC patients with more complex comorbidities because they were more accessible and provided whole-person care [30–32].

**Fig. 1:**
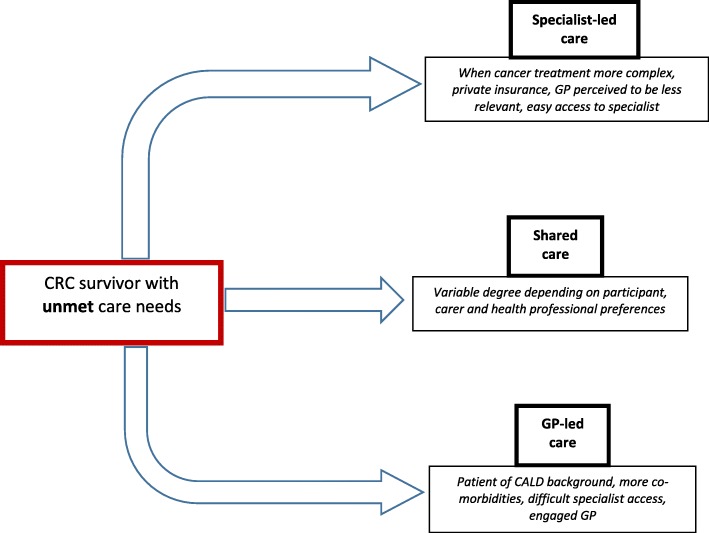
Factors influencing coordination pathways used by CRC survivors

In the absence of formal processes for sharing care, participants in this study described unclear expectations, communication pathways, implementation processes and integration of health care. They adopted a flexible approach using existing processes to seek help primarily from specialists, GPs or both, using informal shared care pathways. This appears to be a dynamic process depending on patient, GP and specialist preferences and taking into account particular health needs and difficulties with access and communication. This is summarised in Fig. 1, which describes care pathways used by CRC survivors according to their care needs, CALD status, and patient and health provider preferences. A recent study of care pathways used by Aboriginal and Torres Strait Islander cancer survivors also concluded that “one size does not fit all” [33].

Non-English-speaking participants had greater difficulty communicating with specialists, and understanding follow-up arrangements. They relied on bilingual GPs following CRC surgery to coordinate care, provide health information, follow-up results, assist with navigating the health care system, and provide emotional support. Bilingual GPs have been previously acknowledged to play a key role in promoting cancer screening [34], presumably because of shared language and cultural insights, but to our knowledge this is the first study describing the role of bilingual GPs in the care of CALD cancer survivors. Data on the number of bilingual GPs in Australia is limited, but one study showed 15.5% of 206 randomly-selected Australian GPs used a language other than English in their consultations [35]. Anecdotally, patients sometimes have long wait times or distances to travel to find a GP who speaks their language.

Findings from our study suggest the cultural and linguistic background of the CRC survivor can influence care pathways. Identifying non-English-speaking cancer survivors from a different cultural and linguistic background could be used to assist in determining the optimal shared care model, as well as factors such as cancer type, stage and comorbidities that are usually used for risk stratification of cancer survivors [36, 37]. Greater effort should be made by specialist services to share care with the bilingual GP consulted by the CALD CRC patient.

### Implications for research and/or practice

Findings from this study reinforce recommendations to include GPs in the care of cancer patients and to support these GPs with the training, resources and pathways to access specialist advice when needed [34]. This study highlights the role of the bilingual GP in translating information and assisting CALD CRC survivors in navigating the health care system. Cancer services could take a more pro-active role to inform patients on the role of the GP in cancer care, including coordination of care, providing continuity, surveillance, cancer prevention, care for emotional needs and for other medical conditions (3). This would involve specific instructions to see the GP for follow-up [38] and clarification of care pathways [39] particularly when the patient comes from a CALD background and is more likely to experience barriers to care because of difficulties with communication and coordination of care. Online localised health pathways [40] are currently being developed which may help clarify expectations, communication and implementation of shared care for CRC survivors. These care pathways are primarily intended for GPs within each health district, but information for patients is being added, and in the future could include patient information in community languages. Proposed shared care plans [9, 41, 42] that include information for the patient, carers and GP would be particularly useful for patients with complex needs and those from CALD backgrounds. Multicultural state-wide health services could assist in connecting cancer survivors with support groups and health services accessible for CALD groups. Further research is required concerning what support is required by bilingual GPs to assist them in caring for CALD cancer survivors.

### Strengths and limitations

This study provides an important insight into the under-researched area of CALD cancer survivorship care. Strengths of the study included participant recruitment from community settings with a broad range of GP and specialist levels of engagement in their care. Interviews with non-English-speaking participants were conducted in their own language by researchers fluent in that language, thus respecting the voice of the informant. This study only included two CALD groups with small numbers in each group, hence the study findings may not be generalizable to other cultural groups. Nevertheless, similar experiences were described by both Spanish and Vietnamese groups. One of the participants had had his initial cancer treatment up to 8 years prior to the study, which may have affected his recollections of post-surgical care, however no substantial changes to the health system have been made since his treatment and his experiences were similar to that of other CRC survivors. Our methodology relied on patient perspectives that limited our ability to independently verify and evaluate the forms of shared care they described. Perspectives of cancer specialists, GPs and other health professionals caring for the participants were not obtained but may have provided additional insights. Further research seeking their perspectives is in progress. Our recruitment strategy may have sampled CRC survivors who were more engaged in follow-up care, reflecting the difficulty engaging more marginalised groups. Patient recruitment from hospital outpatient clinics may also have provided different perspectives on shared care for CRC. Care pathways for CRC survivors within the Australian health care system will differ from those in other countries, however with increasing globalisation and migration the specific care needs of patients from CALD groups and the role of the bilingual GP will be important to consider in future research.

## Conclusions

Non-English-speaking CRC survivors report experiencing similar barriers to care as English-speaking CRC survivors, but experience greater informational and communication needs. Both non-English-speaking and English-speaking CRC survivors describe a blend of specialist-led, GP-led and informal shared care pathways, depending on the complexity of their care needs and perceived engagement and accessibility of the health professionals. Because of the additional challenges navigating care arrangements and the absence of more formal care coordination so far, non-English-speaking CRC survivors tend to rely on family members and bilingual GPs to coordinate their care following surgery. The development of formal shared care plans and localised health pathways should include communication of what CRC survivors can expect from the GP, specialists and other health professionals, particularly targeted at those with complex needs including those from CALD backgrounds.

## Additional file

Additional file 1: Colorectal cancer participant interview guide. (PDF 674 kb)

## Abbreviations

CALD: Culturally and linguistically diverse
CRC: Colorectal cancer
GP: General Practitioner
USA: United States of America
