# Supplementary material for: Perceptions of shared care among survivors of colorectal cancer from non-English-speaking and English-speaking backgrounds: a qualitative study
Source: BMC Fam Pract. 2018 Jul 30;19:134. doi: 10.1186/s12875-018-0822-6 (PMC6066922; doi:10.1186/s12875-018-0822-6)
Supplement: Supplementary file 1 — Colorectal cancer participant interview guide. (PDF 674 kb) [file 12875_2018_822_MOESM1_ESM.pdf]

Patient perspectives on shared care after colorectal cancer surgery

Interview guide

A: INTRODUCTION

(spoken by INTERVIEWER following consent process)

Researchers at the University of Western Sydney would like to help improve the care of patients who have had surgery for colorectal cancer. This study aims to help us to understand the experiences of patients after leaving hospital, and how the different health professionals worked together to care for them.

As part of the research, we are interviewing people from different cultural backgrounds who live in South West Sydney.

We are very keen to hear from you about your experience after your surgery and what worked well for you and what didn't. I would like to ask you a few questions but you are free to answer or not as you prefer. You can also stop the interview at any time if you wish. With your permission, I would like to take notes and record this interview, but what you say will remain confidential to the research team. Your name will not be used in any report without your permission and we will not publish what you have told us in any way that can identify you or your health service. If anything comes up that upsets you in any way we can always stop the interview and ask your family doctor to arrange further support for you.

This interview will last for between 45 – 60 minutes. Are you happy to continue with the interview? Do you have any questions before we begin?

B: PATIENT NARRATIVE ABOUT CANCER DIAGNOSIS

1. Tell me your story from the beginning. How did you find out you had cancer?
   Prompts:
   - What signs were there that something was wrong?
   - Who did you see about it?
   - What did they suggest?
   - What happened next?

C: PATIENT NARRATIVE ABOUT EXPERIENCES AFTER SURGERY

2. Tell me your story about what happened after you left hospital following the surgery

   Prompts:
   - When did you have your operation for colorectal cancer?
   - Can you tell me about the type of surgery you had?

   a. What sorts of complications, if any, did you experience after you got home?
   b. How did you get the help you needed once you left hospital?
   c. What kinds of things made it hard for you to get the help you needed? Prompts as required: trouble with arranging appointments/ transport/ cost
   d. What kinds of things made it easier for you? Support at the time of the consultation? Translation services? Community organisations?
   e. Please tell me about your experience finding the information you needed?

3. What treatment is being planned for you next?

D: COORDINATION OF CARE

4. From your point of view, how well do you think the GP, surgeon and other health professionals worked together as a team?

   Prompts:

   a. Tell me a bit about the different people in your health care team. Who was involved in caring for you after you left hospital? Further prompts as required: surgeon/ GP/ oncologist/allied health/nursing/ counselling or psychologist/ cancer clinic complementary medicine/ Chinese medicine
   b. Which one do you feel was the main professional responsible for coordinating your care?
   c. How well did they communicate with one another? Prompts: How did they exchange information about you and your needs? How did they work out what each other was doing?
   d. Can you tell me about someone who was particularly helpful in coordinating/ arranging your care?

      Prompts:
      - What did they do that you found useful?
      - What longer term health needs did you have after the operation? Prompts: preventive health, emotional support, lifestyle advice, surveillance for cancer recurrence, medical problems unrelated to the cancer? Which health professionals would have been most useful to deal with these needs?

E: A LITTLE BIT ABOUT YOU

I would now like to ask you some questions about yourself.

5. What is your country of birth?
   ____

If not born in Australia: How many years have you lived in Australia?__________________________

6. What language do you speak at home? ____
7. Which age bracket do you belong to? 30-40 40-50 50-60 70-80 80 and above
8. Did you go through the public hospital system or the private system?

F: CONCLUSION

9. How are you going now? Is there anything else you would like to add that might be helpful for the health professionals so they can look after people better following their bowel cancer operation?

10. Do you have any questions for me?

11. Would you like to receive a transcript of the recording to check, or a summary of our findings? If so, could you please give me your address so we can send it to you. Thank you for your time today. I appreciate your coming to talk to me....

NOTES:

Thank participant for time and cooperation.

SHARED CARE: "The joint participation of primary care and specialist doctors in the planned delivery of care for people with long-term conditions, underpinned by enhanced information exchange..." *Hall SJ, Samuel LM, Murchie P. Toward shared care for people with cancer: developing the model with patients and GPs. Family Practice 2011; 28:554-564*
